# Supplementary material for: Lack of Spontaneous and Adaptive Resistance Development in Staphylococcus aureus Against the Antimicrobial Peptide LTX-109
Source: Antibiotics (Basel). 2025 May 11;14(5):492. doi: 10.3390/antibiotics14050492 (PMC12108193; doi:10.3390/antibiotics14050492)
Supplement: Supplementary file 1 [file antibiotics-14-00492-s001.zip › antibiotics-3607020-supplementary.pdf]

Supplementary data

Supplementary Figures:

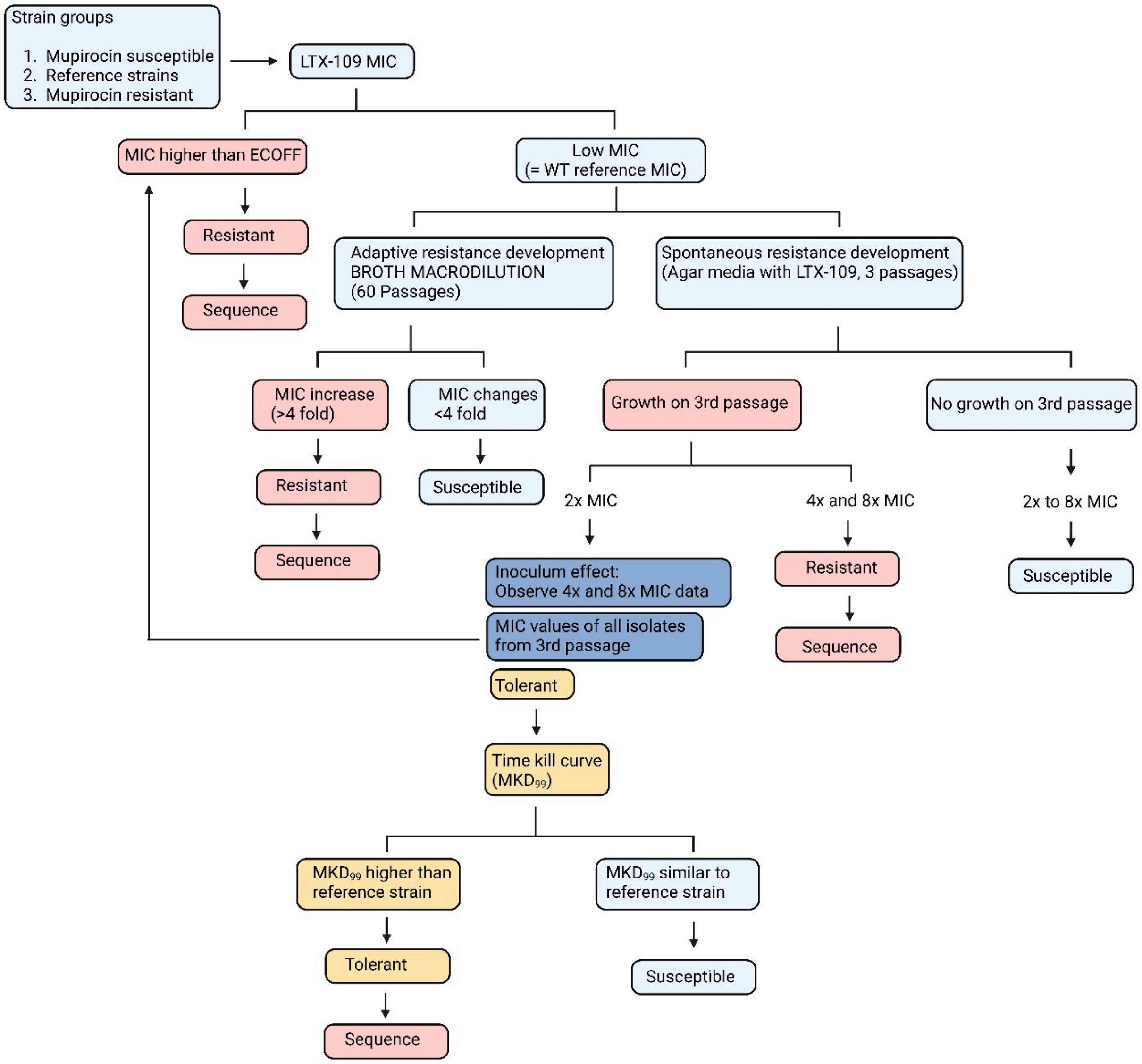

Figure S1. Flowchart depicting the experimental framework used in this study to distinguish susceptible and resistant phenotypes. Strains with MIC higher than wild type reference strain were deemed resistant. Strains with MIC values within the range of wild type reference strains were subjected to resistant development assays, including adaptive (ARD) and spontaneous (SRD) resistant development assays. In ARD performed by broth microdilution (MAC assay), strains that showed > 4-fold increase in MIC during 60 passages were considered resistant, while changes within 4-fold from the initial MIC were considered as natural variations, in the line with EUCAST's MIC evaluation guidelines [1]. For SRD,

resistant was assessed by culturing strains on agar media containing 2x-, 4x- and 8x-MIC anti-microbial over three successive passages. A third passage growth on 4x- and 8x-MIC was considered resistant. Third passage growth on 2x-MIC was scrutinized to exclude: (1) inoculum effect (by considering observations on 4x- and 8x-MIC growth) [2]; (2) Confirm resistance development by evaluating MIC values of all isolates from 3rd passage; as well as considering natural biological variations in MIC values which might influence the true 2x-MIC concentration (again by considering observations on 4x- and 8x-MIC growth) [1]; and (3) tolerance against antimicrobial. This was determined by measuring minimum duration for killing 99% of the population (MKD_{99}) using time-kill curve assays [3]. Strains with MKD_{99} values higher than reference strain were considered as tolerant, otherwise susceptible. Sequencing was planned exclusively for the strains classified as resistant and tolerant. Red-colored boxes indicated the potential need for genome sequencing which was not necessarily due to the lack of phenotypic resistance development.

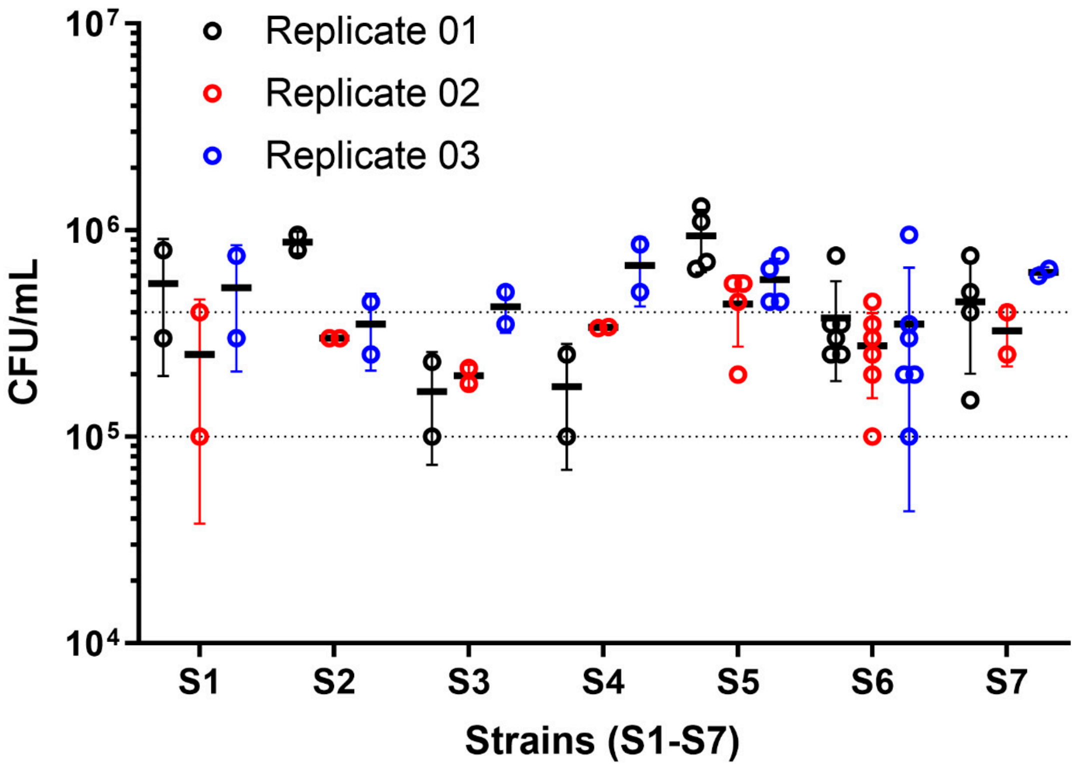

Figure S2. Figure representing BMD assay inoculum. Initial CFU/mL (Y-axis) of strains S1-S7 (X-axis) that were used as inoculums in BMD assay for determining MIC values of strains S1-S7. CFUs were counted at least twice for each biological replicate; each circle represents an individual count. Mean and SD are represented as horizontal and vertical lines, respectively. Dashed horizontal lines on Y-axis represent aimed CFU/mL values between $1x10^5$ to $4x10^5$/mL.

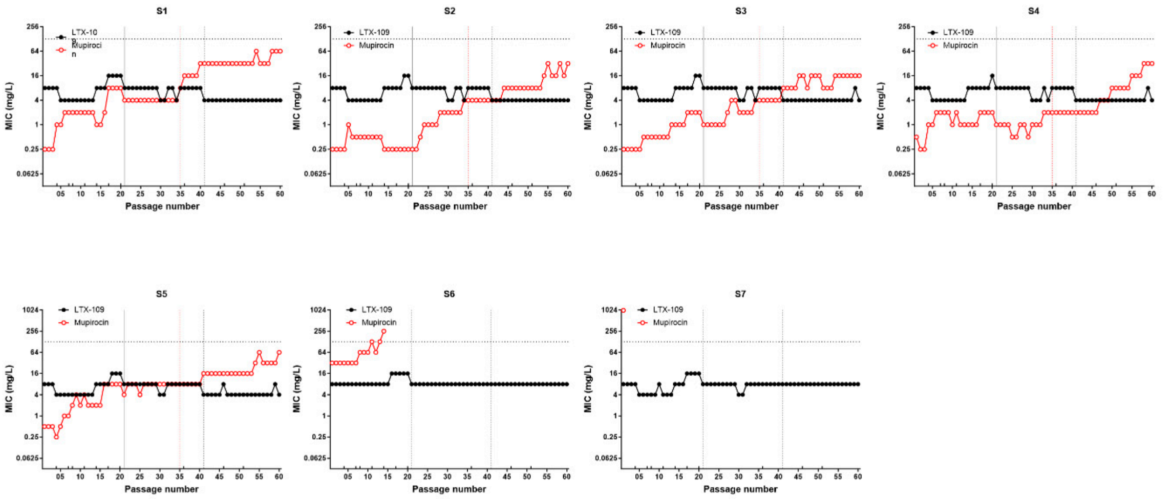

Figure S3. Figure representing MAC assay MIC values over 60 serial passages. MAC assay MIC (mg/L, Y-axis) values of LTX-109 (black), and mupirocin (red) for strains S1-S7 during 60 serial passages (X-axis). MIC value of each passage is represented as a circle, dashed horizontal line on Y-axis represents the cut-off MIC value of 128 mg/L. Vertical lines on X-axis represent the use of a new antimicrobial stock solution (solid line: both LTX-109 and mupirocin, dashed black line: LTX-109 alone, and dashed red line: mupirocin alone).

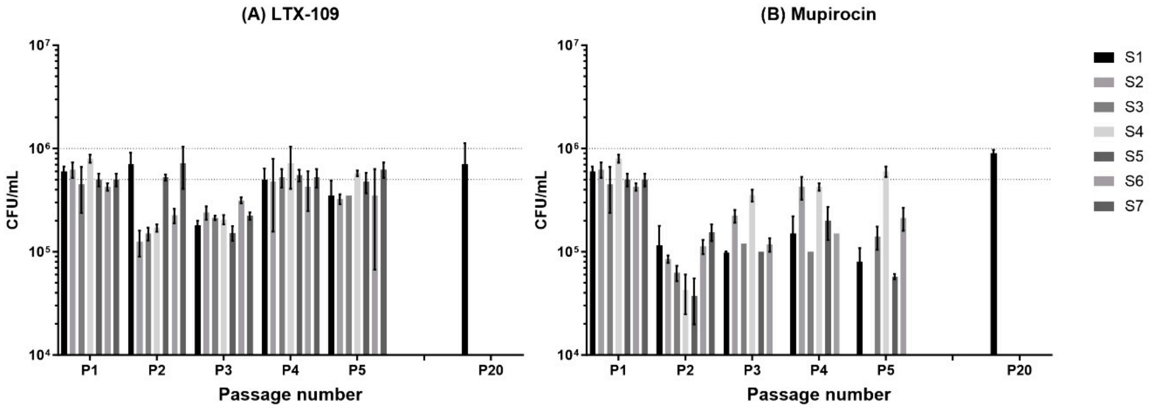

Figure S4. Figure representing MAC assay inoculum. CFU/ml (Y-axis) of strains S1-S7 that were used as inoculums in MAC assay for selected passages (X-axis). CFUs were counted at least twice for each biological replicate; Mean and SD are represented as vertical bars and vertical lines, respectively. Dashed horizontal lines on Y-axis represent an aimed CFU/mL values between 0.5-1.0x10^6/mL.

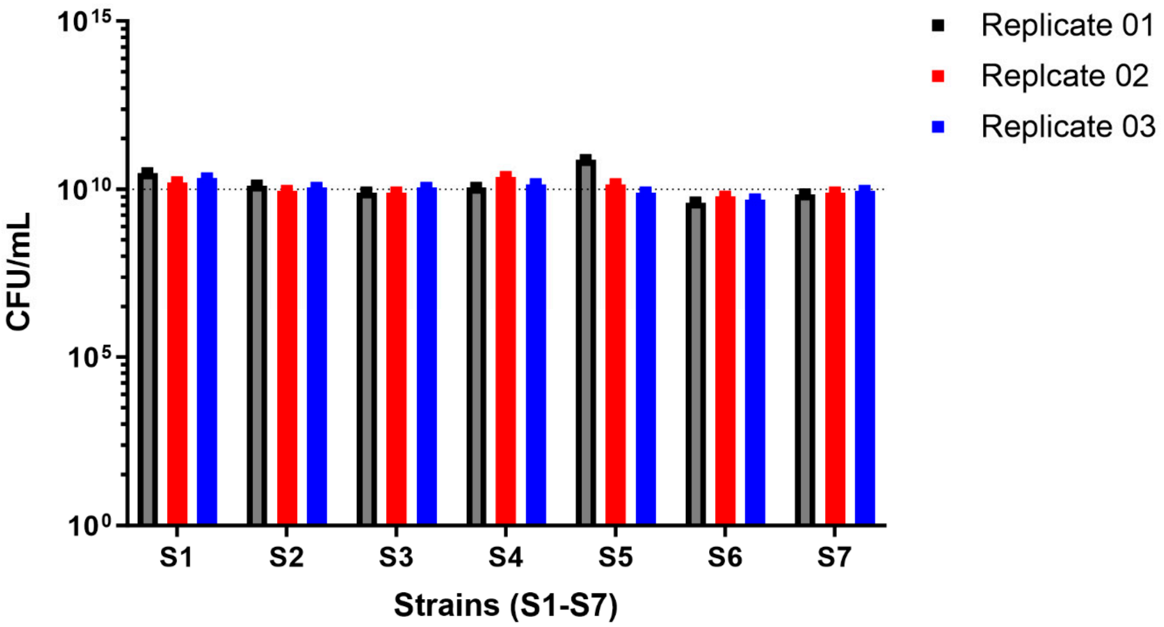

Figure S5. Figure representing SRD assay inoculum. CFU/ml (Y-axis) of strains S1-S7 (X-axis) that were used as inoculums in spontaneous resistance determination (SRD) assays. CFUs were counted at least twice for each biological replicate; Mean CFU/mL is represented as bars. Dashed horizontal lines on Y-axis represent an aimed CFU/mL of $1x10^{10}$/mL.

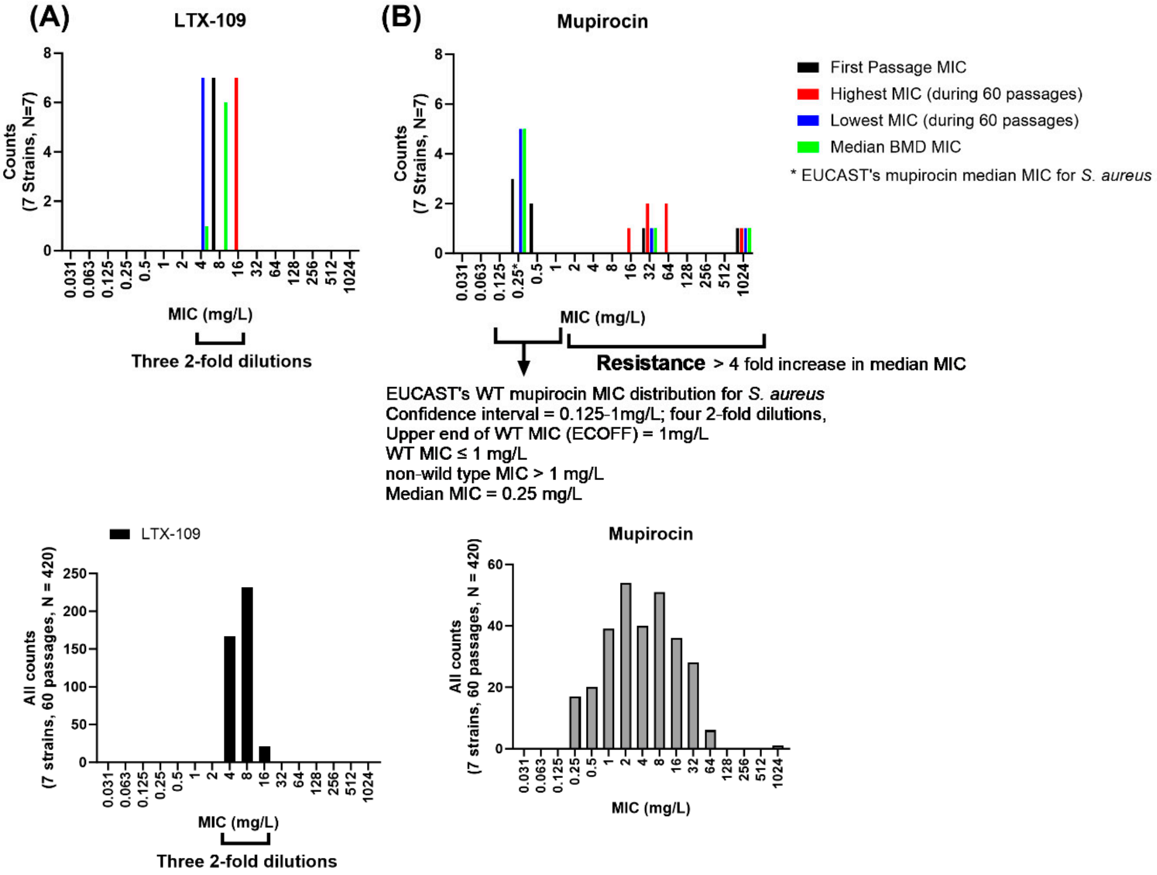

Figure S6. Figure representing changes in MIC vales over 60 serial passages. Changes in MIC values of strains S1-S7 during 60 passages for LTX-109 (A) and mupirocin (B). Y- axis represents numbers of counts and x-axis represents MIC values (mg/L). Top row shows evolution of MIC values (Highest MIC) and fold changes (Highest and lowest MIC) in each strain in comparison to first passage and median BMD MIC (N=7). Bottom row shows all counts of MIC value obtained from all 7 strains during 60 passages (N=420). LTX-109 MIC values showed three 2-fold variations, while mupirocin MIC values for susceptible strains increased >4 fold.

Supplementary files:

Table S1. Table containing Broth microdilution (BMD) assay MIC data. Sheet1 (MIC values) contains Summary of BMD assay MIC values of strains S1-S7 (3 biological replicates and 3 technical replicates. Sheet2 (CFU counts at 0h) contains CFU counts of bacterial cultures (strains S1-S7) used to determine MIC values in BMD assay.

Table S2. Table containing Macrodilution (MAC) assay MIC data**.** Sheet1 (Passages 1-60) contains Summary of MAC assay MIC values for 60 passages for strains S1-S7. Sheet2 (passage 71) contains MIC values (mg/L) for strains S1-S7 evolved in presence of mupirocin and LTX-109 for 60 passages and followed by 10 passages without antimicrobials (Passage 71). Sheet3 (CFU_LTX-109) contains CFU count of the inoculums used in serial passages in LTX-109 for selected bacterial strains for selected passages. Sheet4 (CFU_Mupirocin) contains CFU count of the inoculums used in serial passages in mupirocin for selected bacterial strains for selected passages. Sheet5 (Fold changes) contains calculated fold changes and numbers of 2-fold variations from passage-1 MIC values. EUCAST's definitions of WT and ECOFF, resistance development.

Table S3. Table containing Spontaneous resistance development (SRD) data. Counts of resistance colonies appearing on 2x-, 4x- and 8x-MIC LTX-109 agar plates in biological replicates 01 to 03 in spontaneous resistance (SRD) assay. Sheet1 (inoculums CFU) contains CFU counts of inoculums (strains S1 to S7) used for testing spontaneous resistance. Sheet2 to Sheet4 contains counts of resistance colonies appearing on 2x-, 4x- and 8x-MIC LTX-109 agar plates in biological replicates (BR) 01 to 03. Sheet5 contains MIC values of all the isolates recovered after the second streak (or 3rd passage) on 2x-MIC agar plates in SRD assay.

Table S4. Table containing Time kill curve assay data. CFU/ml counts at 0, 10, 30, 60, 120, and 300 minutes from strains S4 incubated with 0x-, 0.5x-, 2x-, and 8x-MIC LTX-109, and S5 incubated with 0x-, 0.5x-, 1x-, 2x-, 4x-, 8x- and 16x-MIC LTX-109. Each sheet contains data from one biological replicate. Percent CFU reduction in presence of 8xMIC LTX-109 was calculated for each biological replicate.

References:

[1] eucast: MIC and zone distributions and ECOFFs n.d. https://www.eucast.org/mic_and_zone_distributions_and_ecoffs (accessed April 25, 2023).

[2] Udekwu KI, Parrish N, Ankomah P, Baquero F, Levin BR. Functional relationship between bacterial cell density and the efficacy of antibiotics. J Antimicrob Chemother 2009;63:745–57. https://doi.org/10.1093/jac/dkn554.

[3] Brauner A, Fridman O, Gefen O, Balaban NQ. Distinguishing between resistance, tolerance and persistence to antibiotic treatment. Nat Rev Microbiol 2016;14:320–30. https://doi.org/10.1038/nrmicro.2016.34.
